# Supplementary material for: Empirical methods for controlling false positives and estimating confidence in ChIP-Seq peaks
Source: BMC Bioinformatics. 2008 Dec 5;9:523. doi: 10.1186/1471-2105-9-523 (PMC2628906; doi:10.1186/1471-2105-9-523)
Supplement: Additional File 1 — A variety of html documents from the USeq web site detailing the available applications, their best usage, output file type descriptions, command line menus, etc. [file 1471-2105-9-523-S1.zip › USeqUserGuides/JavaDoc.zip!/JavaDoc/index-files/index-17.html]

R-Index


|  |  |  |
| --- | --- | --- |
| **Overview** \| Package \| Class \| Use \| **Tree** \| **Deprecated** \| **Index** \| **Help** | |  |
| **PREV LETTER**   **NEXT LETTER** | **FRAMES**    **NO FRAMES**     **All Classes** |  |


A B C D E F G H I J L M N O P Q R S T U V W X Z 

---


## **R**

**randomize(int)** - Method in class expr.CorrelationMaps: **randomize(RandomizeLabels.ArrayName[])** - Method in class trans.main.RandomizeLabels: **randomize(String[], long)** - Static method in class util.gen.Misc: Randomizes the array by permutating indexes and swapping. **randomize(Object[], long)** - Static method in class util.gen.Misc: Randomizes the array by permutating indexes and swapping. **randomize(float[], long)** - Static method in class util.gen.Num: Randomizes the array by permutating indexes and swapping. **randomize(float[][])** - Static method in class util.gen.Num: Randomizes the intensities, not between replicas. **randomizeFirstArray(float[][], long)** - Static method in class util.gen.Num: Randomizes the first array by permutating indexes and swapping. **RandomizeLabels** - Class in trans.main: **RandomizeLabels(float[][], float[][])** - Constructor for class trans.main.RandomizeLabels: **RandomRegions** - Class in trans.anno: Use to generate random regions of a defined length from a file of regions (chrom, start, stop). **RandomRegions(File)** - Constructor for class trans.anno.RandomRegions: **RandomRegions(File, File)** - Constructor for class trans.anno.RandomRegions: **randomSample(float[], int)** - Static method in class util.gen.Num: Returns an array of float[numToSample] populated with randomly picked values from x. **rangeCheck(double, StringBuffer)** - Method in class trans.qc.StatFlag: Checks min max to see if value is contained withing, adds a 'Warning' line to the StringBuffer if so. **rank(float[])** - Method in class util.gen.SpearmanCorrelation: **RankedFloatArray** - Class in util.gen: **RankedFloatArray(boolean, float[])** - Constructor for class util.gen.RankedFloatArray: **rankedIntersection(GenomicRegion[])** - Static method in class trans.graphics.RankedSetAnalysis: Calculates the number of hits in the ranked matched list. **RankedSetAnalysis** - Class in trans.graphics: Performs set analysis on genomic regions with a visual display. **RankedSetAnalysis(String[])** - Constructor for class trans.graphics.RankedSetAnalysis: **RankedSetDrawFrame** - Class in trans.graphics: Frame for `RankedSetAnalysis`. **RankedSetDrawFrame(RankedSetAnalysis)** - Constructor for class trans.graphics.RankedSetDrawFrame: **RankedSetDrawPanel** - Class in trans.graphics: Panel with the meat for `RankedSetAnalysis` **RankedSetDrawPanel(RankedSetAnalysis)** - Constructor for class trans.graphics.RankedSetDrawPanel: **RankSample** - Class in util.gen: **RankSample(int, float)** - Constructor for class util.gen.RankSample: **RankSampleIndexComparator** - Class in util.gen: Sorts by index, small to large. **RankSampleIndexComparator()** - Constructor for class util.gen.RankSampleIndexComparator: **rankSamplePairs()** - Method in class trans.main.WilcoxonSignedRankTest: Ranks a sorted array of WilcoxonSamplePairs based on absoluteDifference. **rankSamples()** - Method in class trans.main.WilcoxonRankSumTest: Ranks a sorted array of WilcoxonSamples based on value. **rankSamples(RankSample[])** - Method in class util.gen.SpearmanCorrelation: Ranks a sorted array of RankSample based on value. **RankSampleValueComparator** - Class in util.gen: Sorts by value, small to large. **RankSampleValueComparator()** - Constructor for class util.gen.RankSampleValueComparator: **ratio(double[], double[])** - Static method in class util.gen.Num: Returns the ratio of each pair. **ratio(float[][], float[][])** - Static method in class util.gen.Num: Returns the ratio of each pair, averages replicas. **ratio(float[], float[])** - Static method in class util.gen.Num: Returns the ratio of each pair. **ratio(int[], double[])** - Static method in class util.gen.Num: Returns the ratio of each pair. **ratio(int[], int[])** - Static method in class util.gen.Num: Returns the ratio of each pair. **RawDataBean** - Class in cgar.beans: Pointer to raw data file and id in GNomEx. **RawDataBean()** - Constructor for class cgar.beans.RawDataBean: **Read** - Class in edu.utah.seq.methylation454: Sequence aligned to a reference. **Read(String, String)** - Constructor for class edu.utah.seq.methylation454.Read: example of a header and a seqLine >ETK72AO01CGP6K /amp="I14" /cId="CON\_1" /id="ETK72AO01CGP6K" /qCd="24-45" /lCp="5" /rCd="24-45" /rRg="24-56" /mFc="60" /str="1" 23 AA-T-AA-T-AAA-AA-TTTT-A-G-A-TA-TG 1452 **READ\_LENGTH\_TAG** - Static variable in class edu.utah.seq.parsers.BarParser: **ReadAlignParser** - Class in edu.utah.seq.methylation454: **ReadAlignParser(File, File)** - Constructor for class edu.utah.seq.methylation454.ReadAlignParser: **readBarFile(File, boolean)** - Method in class edu.utah.seq.parsers.BarParser: Reads a bar file and sets the appropriate fields in the BarParser. **readBinary(File)** - Static method in class util.bio.annotation.Coordinate: Reads a binary Coordinate[] file. **readBinaryInt2Array(File)** - Static method in class util.gen.Num: Reads a binary int[][2] array file. **readBinarySequence(File)** - Static method in class util.bio.seq.Seq: Reads a binary sequence file returning gatc or n, lower case. **readOutHash()** - Method in class util.bio.parsers.UCSCGeneModelTableReader: To test your hash. **readSimpleGrBarFile(File)** - Static method in class trans.misc.Util: This is a complete hack of Gregg Helt's IGB code to parse bar files. **readTagValPairs(DataInput, int)** - Method in class edu.utah.seq.parsers.BarParser: Method for parsing tag values from a binary stream. **readTagValPairs(DataInput, int)** - Static method in class trans.misc.Util: Methodfor parsing tag values from a binary stream. **ReadZip** - Class in util.gen: **ReadZip()** - Constructor for class util.gen.ReadZip: **ReagentBean** - Class in bioroot: **ReagentBean()** - Constructor for class bioroot.ReagentBean: **reallyRedrawAnnotationPanel(AnnoSpecParams)** - Method in class gata.menu.GATAFrame: **REDigest** - Class in util.bio.digest: StandAlone class to perform a restriction digestion on a sequence, a hack, uses as a template. **REDigest()** - Constructor for class util.bio.digest.REDigest: **redrawAlignPanel()** - Method in class gata.menu.GATAFrame: **redrawAnnotation()** - Method in class gata.geneGlyphs.Annotation: **redrawAnnotationPanels()** - Method in class gata.menu.GATAFrame: basically draws annotation twice, once to estimate the size, the second time for real **redrawGlyphPanel(AnnoSpecParams)** - Method in class gata.plotter.AlignPanel: **Reference** - Class in edu.utah.seq.methylation454: **Reference(String)** - Constructor for class edu.utah.seq.methylation454.Reference: Alignment file line for a reference. **reformatSeq(int[][], String, String)** - Static method in class gata.plotter.ConservedSeqs: Reformats a sequence given and int[][] containing start stops **RefSeqAnnotationParser** - Class in util.bio.parsers: Parser for pulling gene annotations into a UCSC table format for Sodalis. **RefSeqAnnotationParser()** - Constructor for class util.bio.parsers.RefSeqAnnotationParser: **Region** - Class in trans.anno: Represents a genomic region. **Region(String, int, int, String)** - Constructor for class trans.anno.Region: **RegionComparator** - Class in trans.anno: **RegionComparator()** - Constructor for class trans.anno.RegionComparator: **Regions2Intervals** - Class in trans.misc: Converts binding regions to intervals setting the binding region as the best window and adds 2kb on either side. **Regions2Intervals()** - Constructor for class trans.misc.Regions2Intervals: **relativeDifference(double, double)** - Static method in class util.gen.Num: Calculates a relative difference. **relativeDifferences(float[], float[])** - Static method in class util.gen.Num: Calculates relative differences. **relativeDifferences(float[][], float[][])** - Static method in class util.gen.Num: Returns the relative difference of each pair, averages replicas. **relativeDifferenceToLog2Ratio(float[])** - Static method in class util.gen.Num: Converter **relativeDifferenceToLog2Ratio(double)** - Static method in class util.gen.Num: Converter **removeAdjacentDuplicates()** - Method in class trans.tpmap.TPMapDuplicateFilter: Throws out data lines that are immediately adjacent and have the same sequence. **removeDuplicateMappingOligos(File)** - Method in class trans.tpmap.TPMapDuplicateFilter: Removes any oligo that is found more than once in the bpmap file. **removeExtension(File, String)** - Static method in class util.gen.IO: Removes the extension from each File in the directory. **removeExtension(String)** - Static method in class util.gen.Misc: Removes an extension if found xxx.txt -> xxx. **removeLowOligoWindows(Window[], int)** - Static method in class trans.misc.Util: Removes windows that contain < minNumberOligos. **removeLowQualitySegs(int[][])** - Method in class selex.SeqRead: **removeMaskedRegions()** - Method in class trans.roc.RocWindowScanner: Remove windows that intersect the masked regions. **removeNaNInfinite(double[])** - Static method in class util.gen.Num: Removes any NaN or Infinite **removeOddSizedSegs(ArrayList)** - Method in class selex.SeqRead: **removeParent(StrainBean, HashMap, DBUtil)** - Static method in class bioroot.strain.NewStrain: **removePlasmid(AntibodyBean, HashMap, DBUtil)** - Static method in class bioroot.antibody.AntibodyBase: **removePlasmid(StrainBean, HashMap, DBUtil)** - Static method in class bioroot.strain.NewStrain: **removeRegion(int)** - Method in class trans.roc.ScoreParsedBars: **removeStrain(AntibodyBean, HashMap, DBUtil)** - Static method in class bioroot.antibody.AntibodyBase: **removeStrain(HashMap, PlasmidBean, DBUtil)** - Static method in class bioroot.plasmid.NewPlasmid: **removeZeroValues(float[][], float[][])** - Static method in class util.gen.Num: Removes zero values and their assoicated values from all arrays. **removeZeroValues(float[])** - Static method in class util.gen.Num: Remove zero values from float array. **removeZeroValues(float[][])** - Static method in class util.gen.Num: Provide a float[2][0], will remove zeros from both and there matched pair. **renameCelFiles()** - Method in class trans.main.T2Parameter: Replaces the File pointers to the converted xxx.cela files preserving the input structure. **renameFile(String, String)** - Static method in class bioroot.antibody.AntibodyBase: Copies an old file and renames it to tempRndmWrd\_name. **renameFiles(File, String, String)** - Static method in class util.gen.IO: Renames files containing the toReplace with replaceWith using String.replaceAll(String, String) **RenameRWSResults** - Class in util.apps: Takes the output of RocWindowScanner from the cluster and renames the file **RenameRWSResults()** - Constructor for class util.apps.RenameRWSResults: **renameToACels(File[][])** - Method in class trans.main.T2Parameter: **RenameWords** - Class in bioroot.parsers: **RenameWords(String[])** - Constructor for class bioroot.parsers.RenameWords: **RepeatMaskerToGFF** - Class in util.bio.converters: Converts a RepeatMasker output to GFF annotation. **RepeatMaskerToGFF(String[])** - Constructor for class util.bio.converters.RepeatMaskerToGFF: **replaceEnd(String, String, String)** - Static method in class util.gen.Misc: Attempts to match the end of the word with the match, case insensitive, replaces it if found, otherwise returns null. **replaceQuotations()** - Method in class util.apps.HouseBean: **ReplaceWindowScores** - Class in trans.main: **ReplaceWindowScores(String[])** - Constructor for class trans.main.ReplaceWindowScores: **reset()** - Method in class bioroot.Feature: **resetFields()** - Method in class util.bio.parsers.MultiFastaParser: **resetForm()** - Method in class gata.aligner.AlignerInputPanel: **resetOrderNumber(HttpServletRequest)** - Method in class bioroot.TableHeading: Sets orderNumber from parameter provided, the Param can be converted to an int that is >0. **resetStartStop()** - Method in class trans.roc.MethodTester: Scans the bins to determine the optimal start and stop for the next round. **restrictionMap(String, Enzyme[])** - Static method in class util.bio.digest.REDigest: Runs thru an enzyme array attempting to cut a particular sequence **restrictionMapHTML(String, Enzyme[])** - Static method in class util.bio.digest.REDigest: Returns an arraylist of enzymes found to cut the sequence **restrictionMapList(String, Enzyme[])** - Static method in class util.bio.digest.REDigest: Restriction map a sequence. **reverse(String)** - Static method in class util.gen.Misc: Reverses a String using a StringBuilder. **reverseAntibodyBeans(AntibodyBean[])** - Static method in class bioroot.antibody.AntibodyBase: Reverses the order of an array of PlasmidBeans. **reverseCompDNA(String)** - Static method in class meme.MemeParser: takes DNA seq and reverse comps it ambiguous symbols OK. **reverseCompDNA(String)** - Static method in class util.bio.xamer.XamerCounter: **reverseComplementDNA(String)** - Static method in class util.bio.seq.Seq: Takes a DNA seq and reverse comps it, ambiguous symbols OK. **reverseOligoBeans(OligoBean[])** - Static method in class bioroot.oligo.OligoBase: Reverses the order of an array of OligoBean. **reversePlasmidBeans(PlasmidBean[])** - Static method in class bioroot.plasmid.PlasmidBase: Reverses the order of an array of PlasmidBeans. **reverseStrainBeans(StrainBean[])** - Static method in class bioroot.strain.StrainBase: Reverses the order of an array of StrainBeans. **RipInparanoidResults** - Class in util.bio.parsers: **RipInparanoidResults()** - Constructor for class util.bio.parsers.RipInparanoidResults: **RipMGIOrthologyResults** - Class in util.bio.parsers: **RipMGIOrthologyResults()** - Constructor for class util.bio.parsers.RipMGIOrthologyResults: **RocWindow** - Class in trans.roc: For holding information about a region for ROC analysis. **RocWindow(String, int, int, double)** - Constructor for class trans.roc.RocWindow: **RocWindowScanner** - Class in trans.roc: Scans a window array over a range of thresholds and intersects with a list of positives to generate the false positive rate, true positive rate, and false discovery rates. **RocWindowScanner(String[])** - Constructor for class trans.roc.RocWindowScanner: **RotateCelFile** - Class in trans.cel: **RotateCelFile()** - Constructor for class trans.cel.RotateCelFile: **rotateCelFile(File, int)** - Static method in class trans.cel.RotateCelFile: Rotates a text version cel file 90 degrees clockwise. **rotateClockwise(float[][])** - Static method in class util.gen.Num: Rotates a square matrix 90 degrees Clock Wise? **rotateCounterClockwise(float[][])** - Static method in class util.gen.Num: Rotates a square matrix 90 degrees CounterClockWise? **round(double[], int)** - Static method in class util.gen.Num: Rounds a double[] based on number of desired decimals, ie 1 = x.x, 2 = x.xx limited to 6 **round(double, int)** - Static method in class util.gen.Num: Rounds a double based on number of desired decimals, ie 1 = x.x, 2 = x.xx limited to 6 **run()** - Method in class gata.aligner.AlignerThread: **runMummerExactMatcher(File)** - Method in class trans.tpmap.MummerMapper: Runs MUMMER to find perfect matches

---


|  |  |  |
| --- | --- | --- |
| **Overview** \| Package \| Class \| Use \| **Tree** \| **Deprecated** \| **Index** \| **Help** | |  |
| **PREV LETTER**   **NEXT LETTER** | **FRAMES**    **NO FRAMES**     **All Classes** |  |


A B C D E F G H I J L M N O P Q R S T U V W X Z 

---
